# The Role of Mutations, Addition of Amino Acids, and Exchange of Genetic Information in the Coevolution of Primitive Coding Systems

**DOI:** 10.3390/ijms26157176

**Published:** 2025-07-25

**Authors:** Konrad Pawlak, Paweł Błażej, Dorota Mackiewicz, Paweł Mackiewicz

**Affiliations:** Department of Bioinformatics and Genomics, Faculty of Biotechnology, University of Wrocław, ul. Joliot-Curie 14a, 50-383 Wrocław, Poland; konrad.pawlak@uwr.edu.pl (K.P.); dorota.mackiewicz@uwr.edu.pl (D.M.); pawel.mackiewicz@uwr.edu.pl (P.M.)

**Keywords:** amino acid, codon, codon usage, genetic code, mutations, selection

## Abstract

The standard genetic code (SGC) plays a fundamental role in encoding biological information, but its evolutionary origins remain unresolved and widely debated. Thus, we used a methodology based on the evolutionary algorithm to investigate the emergence of stable coding systems. The simulation began with a population of varied primitive genetic codes that ambiguously encoded only a limited set of amino acids (labels). These codes underwent mutation, modeled by dynamic reassignment of labels to codons, gradual incorporation of new amino acids, and information exchange between themselves. Then, the best codes were selected using a specific fitness function *F* that measured the accuracy of reading genetic information and coding potential. The evolution converged towards stable and unambiguous coding systems with a higher coding capacity facilitating the production of more diversified proteins. A crucial factor in this process was the exchange of encoded information among evolving codes, which significantly accelerated the emergence of genetic systems capable of encoding 21 labels. The findings shed light on key factors that may have influenced the development of the current genetic code structure.

## 1. Introduction

The standard genetic code (SGC) was discovered in the 1960s [1,2]. Mathematically speaking, the SGC is a map between the set of 64 possible codons and 20 amino acids plus the stop coding signal [3], which enables the transmission of genetic information from the nucleic acids into proteins. This important finding immediately opened a discussion about potential mechanisms of coding system emergence [4] because the SGC is nearly universal, with rare exceptions of alternative genetic codes [5,6]. Some archaea and bacteria lack certain aminoacyl-tRNA synthetases, i.e., asparaginyl- or glutaminyl-tRNA synthetase, but they still manage to incorporate all 20 amino acids using indirect biosynthetic pathways—attaching a non-cognate amino acid to tRNA and then converting it to the correct one [7].

The main questions posed to the problem of SGC emergence are related to decisive factors that shaped its present structure. In order to answer this problem, several hypotheses have been proposed, namely stereochemical, coevolution, and adaptive, and physiochemical hypotheses [8,9,10,11,12,13]. These explanations point to different factors and scenarios of genetic code evolution. The stereochemical theory proposes that codon–amino acid assignments arose from direct physicochemical interactions between amino acids and specific codons/anticodons or nucleotide aptamers. Coevolution theory suggests that the code expanded alongside biosynthetic pathways, with new amino acids inheriting codons from metabolic precursors. The adaptive theory argues that the code evolved to minimize mutations and translation errors, by clustering codons for amino acids with similar physicochemical properties.

However, it is not inconceivable that the evolution of the standard genetic code, since the beginning, was a trade-off between several factors that together influenced the present structure of the SGC [13,14,15]. For example, recent analyses by Caldararo and Di Giulio [16] support coevolution theory, proposing that the addition of amino acids to the SGC followed their relationships in biosynthetic pathways, playing a decisive role in structuring the code—specifically organizing the rows of the SGC table. In contrast, the allocation of amino acids to its columns was optimized based on the partition energy, which quantifies residue interaction energetics relative to a solvent reference state. This column-wise optimization likely arose from strong selection pressures favoring efficient protein folding and enzymatic catalysis.

Following adaptive theory, one of the important features considered in the evolution of the codes was a tendency to minimize errors in the translation process and substitutions of coded amino acids [17,18,19,20]. The mathematical analysis of the genetic code also demonstrated its robustness to errors of reading genetic information [21,22]. However, the SGC turned out to not minimize errors better than its natural alternatives [23] and codes optimized by genetic algorithms [24,25,26,27,28,29]. Thus, it seems that the optimization of mutational pressure at the replication level had to supplement the properties of the genetic code to reduce the consequences of harmful substitutions [30,31,32,33].

Some authors pointed out the role of the horizontal gene transfer in the genetic code emergence [34,35]. Based on their studies and considerations, they concluded that the exchange of genetic information was important for developing the universality and optimality of the genetic code.

It was also postulated that genetic information in the early genetic code was not precisely coded and the high translational noise was then minimized during the code evolution [9,36]. The simulation of SGC emergence from the ambiguous assignments of codons to the system of highly unambiguous coding patterns showed that a stable system could emerge as a set of codons to which the set of encoded labels, i.e., 20 amino acids and stop coding signals, was unambiguously assigned [37,38]. Interestingly, simulated coding systems have been similar to the SGC in terms of coding redundancies, i.e., blocks of synonymous codons encoding the same amino acid. This feature appeared even when there were no assumptions on the properties of encoded amino acids. However, previous studies did not include the possibility of the simultaneous evolution of coding systems encoding different numbers of labels and the exchange of genetic information between evolving codes.

The idea of coexistence and simultaneous evolution of primitive coding systems was first postulated by Crick [39]. He proposed that the initial phase of code evolution was represented by primitive coding systems, which coded only a few amino acids using a small number of triplets. From these codes evolved intermediate coding systems, in which additional amino acids took over most of the remaining triplets to reduce nonsense codons to a minimum. As a result of this process, the current code with 20 amino acids and a stop translation signal emerged. Although the intermediate coding systems could already code the whole repertoire of these elements, the coding was not likely as precise as in the SGC.

It is highly probable that primordial coding systems are characterized by smaller numbers of coding labels. For example, biochemical and metabolic coevolution theories propose that the earliest amino acids in the code were those readily available through prebiotic synthesis, while others entered the code later via biosynthetic pathways when they gradually evolved. This stepwise addition is supported by the metabolic relationships among amino acids and their codon assignments, showing that the genetic code coevolved with amino acid metabolism [13,40].

Since the early stages of genetic code including many competitive coding systems were not extensively studied, we applied them in a simulation model. We assumed that the initial codes did not code more than seven labels. This agrees with the studies in [41], which postulate that six amino acids, i.e., Gln, Gly, Leu, and possibly Pro, Asp, and Asn, were likely coded by an early genetic code. We also implemented a reduction in the initial high ambiguity of the label-to-codon ascription and errors in codon reading. Additionally, the model assumed the stepwise addition of amino acids to the codes and the transfer of genetic information between the evolving codes. The simulated evolution of various coding systems enabled us to observe the transition from primitive to intermediate coding systems characterized by low ambiguity in the coding of 21 translation signals. The quality of a given coding system was measured by a fitness function *F*, whose value increases as the coding system becomes more capable of encoding all studied labels and as codons more clearly and unambiguously correspond to their respective labels. The optimization of codes was achieved by evaluating the final fitness function after each simulation and adjusting the relative probabilities of selecting each code accordingly. Thereby, our model also follows the assumptions of the adaptive theories assuming the minimization of mutations and translational errors during code evolution. The model aimed to evaluate the significance of three key factors: (i) mutations altering the assignment of amino acids and stop signals to codons, (ii) the progressive incorporation of new amino acids into the code, and (iii) the exchange of genetic information between organisms carrying these codes. The competition among evolving codes mimicked natural selection, providing insights into the forces shaping genetic code evolution.

## 2. Results

### 2.1. The Initial Genetic Codes and Assumptions on Their Evolution

During the simulations, we could distinguish two classes of genetic codes: the primitive genetic codes encoding less than the maximum of 21 labels with high ambiguity and the intermediate genetic codes encoding 21 labels with lower ambiguity. Thus, the standard genetic code should be considered in this context as the final code encoding 21 labels unambiguously.

**Figure 1:**
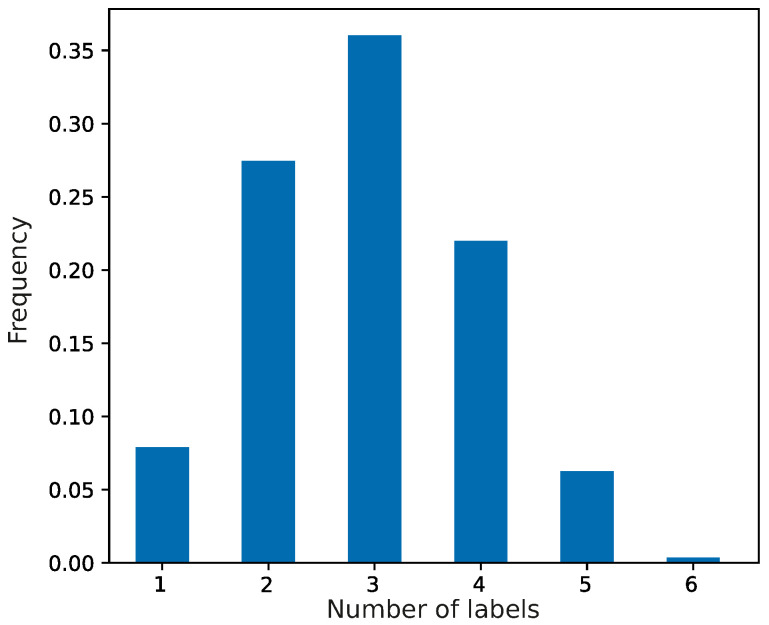
The frequencies of coding systems at the beginning of the simulations for encoding a given number of labels. The coding systems were generated randomly according to the truncated Poisson distribution, so the population of coding systems represents a sample from this distribution.

At the beginning of the simulations, the evolving population of genetic codes was composed of primitive coding systems, which were characterized by random assignments of codons to the set of genetic information. We assumed that each codon of this system could encode no more than seven labels with a given probability. The frequencies of the coding systems are presented in Figure 1. The most numerous were codes that encoded three labels, and the least frequent were those with six labels encoded.

**Figure 2:**
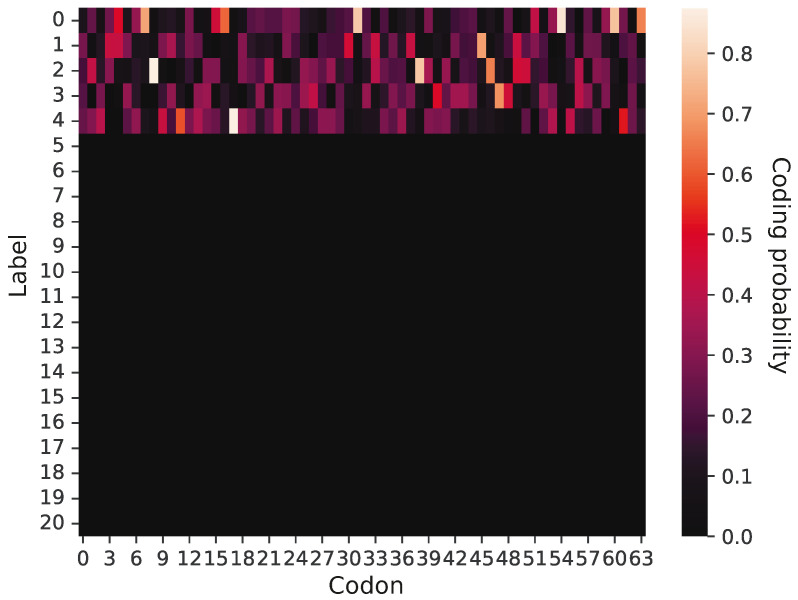
The graphical representation of a randomly chosen primitive genetic code at the beginning of simulation, which encodes five labels. The values of the probability function are depicted through color intensity. The bright colors indicate a high probability that a given codon (x-axis) encodes a selected label (y-axis), whereas dark colors indicate a low coding probability. Since the code contains only five labels; others have a zero coding probability and are represented by black rows.

An example of a primitive coding system is presented in Figure 2, in which 64 potential codons (x-axis) are assigned to five labels (y-axis). According to this graphical representation, the probability that a given codon encodes a label is visualized by color intensity. It is evident that this coding system is ambiguous and does not demonstrate a strong coding pattern; namely, there are no blocks of codons to encode a given label with a high probability. In contrast, a given label can be coded by many codons with a low or moderate probability. This coding system encodes only five labels, so the remaining 16 labels have zero coding probability. However, the primitive genetic codes were evolving under simulation constraints, which allowed them to create more unambiguous assignments of labels to codons as well as acquire new genetic information through the addition of new labels and the exchange of information between other codes. Thereby, the codes could reach the phase of genetic codes including 21 labels.

Moreover, to model an initial inaccuracy of translation, we applied three types of codon reading, $N_{1}$, $N_{2}$, and $N_{3}$ applied together (see the Materials and Methods section for more details). For each reference codon, we identified neighborhood codons that coded a given label and differed in at most one nucleotide position from the reference codon. As a consequence of the high ambiguity, potential protein-coding sequences could be translated into a family of protein sequences instead of a single protein. During each simulation run, all coding systems could acquire new genetic information during the following processes: the mutation of label-to-codon assignment (described by parameter $m_{c}$), the introduction of new coding labels into the coding system (described by parameter $m_{l}$), and the exchange of genetic information between evolving coding systems (described by parameter $m_{e}$). The latter process assumed an interaction between the coding systems transferring genetic information. We ran the simulations under all possible combinations of values for these parameters.

### 2.2. The Course of Genetic Code Evolution

**Figure 3:**
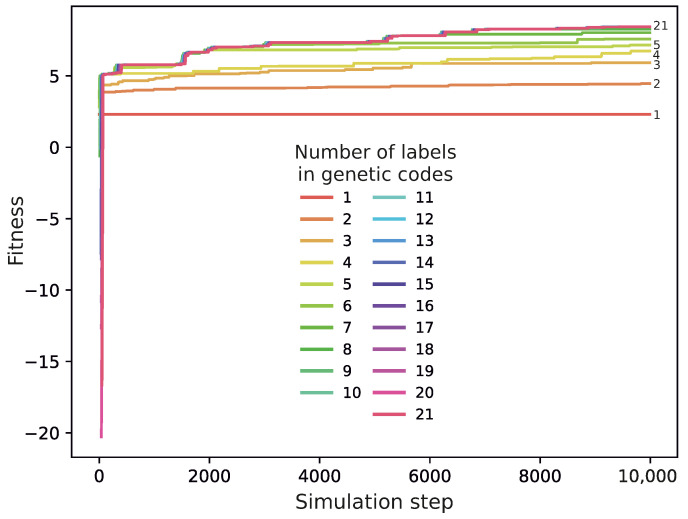
Changes in the average fitness of genetic codes with various numbers of labels during the simulation run. This simulation was conducted under the following parameters: probability of change in the assignment of labels to codons $m_{c}=0.1$, probability of acquisition of new labels with a code $m_{l}=0.01$, and the probability of exchange of genetic information between codes $m_{e}=0.05$.

The fitness function was calculated for each genetic code to assess its quality in terms of potential robustness against translational errors. The reduction in ambiguity was partially driven by this function, which preferred codes with a lower ambiguity. It was also applied to investigate the process of the transformation of the coding systems. As we can see in Figure 3, the average fitness calculated for a group of genetic codes encoding a selected number of labels increases very rapidly already at the early steps of the simulation after these codes emerge. However, the fitness of codes encoding fewer labels saturates more quickly and at lower values than that of codes with a higher number of labels, whose fitness continues to increase for longer and reaches higher levels.

**Figure 4:**
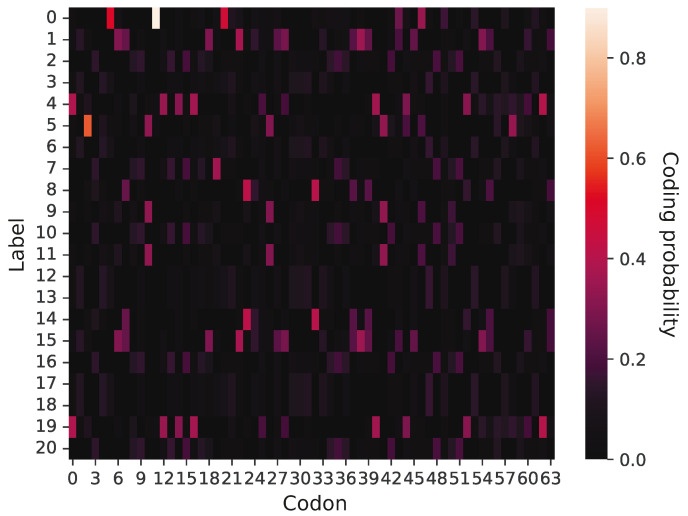
Graphical representation of an intermediate genetic code with 21 labels at the end of the simulation. The labels were encoded with much lower ambiguity than in the primitive codes. The values of the probability function are depicted through color intensity. The bright colors indicate a high probability that a given codon (x-axis) encodes a selected label (y-axis), whereas dark colors indicate a low coding probability.

**Figure 5:**
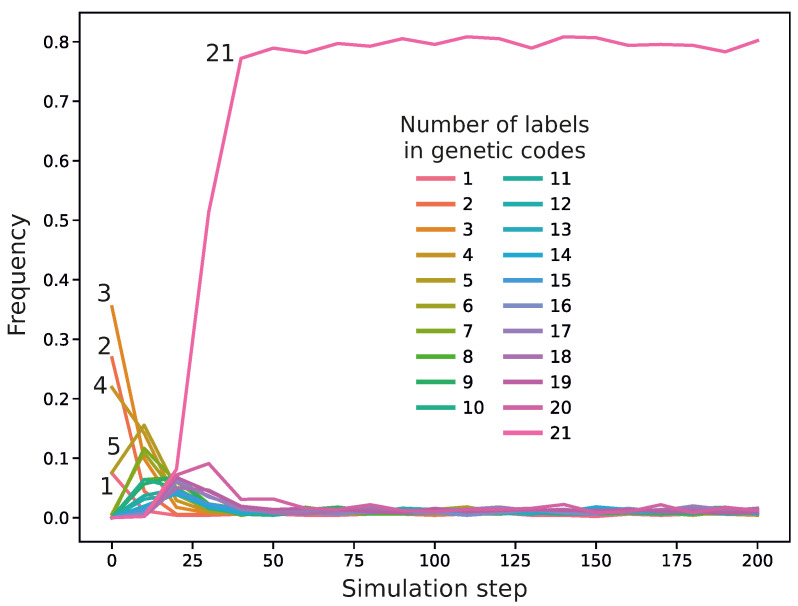
Changes in the frequency of genetic codes with various numbers of labels during the simulation run. This simulation was conducted with the following parameters: the probability of change in the assignment of labels to codons $m_{c}=0.2$, the probability of acquisition of new labels with a code $m_{l}=0.09$, and the probability of exchange of genetic information between codes $m_{e}=0.09$.

The simulations usually ended with the code including 21 labels. An example of this code encoding the full repertoire of genetic information is shown in Figure 4. In this code, we can notice codon blocks encoding genetic labels with high probabilities. For example, codon 0 encodes with a high probability two labels, 4 and 19, whereas codon 41 encodes three labels, 5, 9, and 11. The codes with a larger number of labels usually won the competition with other codes during the simulations. For example, Figure 5 presents the frequency of genetic codes with various numbers of labels in the population of codes during the simulation run. As we can see, coding systems with 21 labels emerged quickly and dominated the evolving genetic codes quite early, already in the 24th simulation step, whereas those with fewer labels, initially abundant, practically disappeared in later simulation steps. We found that in 809 out of 990 simulations, the fraction of the 21-label codes exceeded 50% before the end of the simulations. Moreover, in 792 out of 809 cases, the codes reached this domination before 1000 steps, i.e., within 10% of the whole simulation time. On the other hand, there were 181 simulations in which the codes encoding 21 labels did not reach 50% contribution by the end of the simulations, i.e., 10,000 steps.

### 2.3. The Relationship Between Genetic Code Evolution and Simulation Parameters

In order to estimate the influence of individual factors on genetic code evolution, we analyzed relationships between simulation parameters and properties of evolving coding systems. We observed that in all simulations, the genetic codes encoding 21 labels with lower ambiguity emerged under all studied parameters. However, there were substantial differences in the time of the first appearance of such codes depending on the parameters. In the simulation conducted under constraints $m_{c}=0.2$, $m_{l}=0.1$, and $m_{e}=0.1$, the genetic code emerged after 10 simulation steps, whereas in the cases of $m_{c}=0.7$, $m_{l}=0.01$, and $m_{e}=0.00$, it requires 160 steps. This huge difference is mainly caused by the probability of acquisition of new labels with a code ($m_{l}$), which is obvious, and, interestingly, by the exchange of information between codes ($m_{e}$), which corresponds to the horizontal gene transfer (HGT) between organisms. The slowest evolution of codes encoding 21 labels was observed when the former factor was the smallest and the latter factor was not included. The fastest occurrence was when these two parameters were set for the maximum tested values.

**Figure 6:**
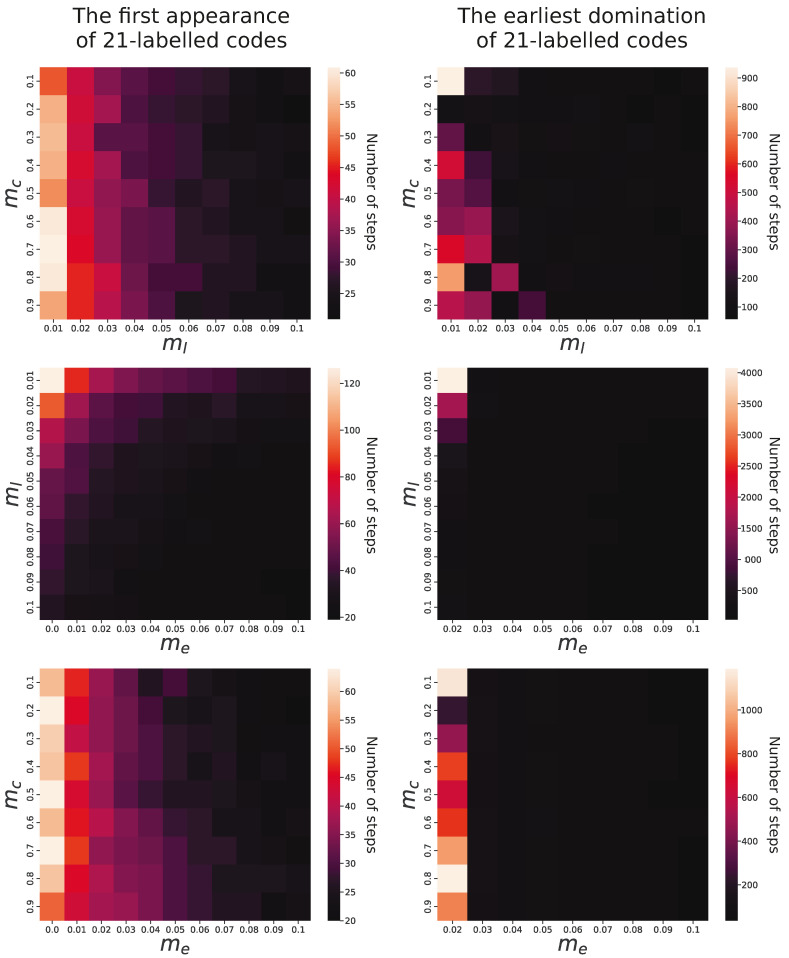
The first appearance and the earliest domination (in the number of steps) of genetic codes encoding 21 labels during simulations in terms of relationships with various combinations of three parameters: the probability of change in the assignment of labels to codons ($m_{c}$), the probability of the acquisition of new labels with a code ($m_{l}$), and the probability of the exchange of genetic information between codes ($m_{e}$). The bright colors indicate the code’s late appearance or supremacy, whereas dark colors indicate an early appearance or dominance.

Pairwise comparisons of the parameters and their influence on the first appearance of genetic codes encoding 21 labels are visualized in the left panel of Figure 6. It is clear that the higher probability of the addition of new labels into evolving codes ($m_{l}$) together with the exchange genetic information ($m_{e}$) substantially facilitated the faster development of the 21-label codes. The probability of change in the assignment of labels to codons ($m_{c}$) did not influence the evolution of the code significantly.

The right panel of Figure 6 presents the relationship of $m_{c}$, $m_{l}$, and $m_{e}$ with the number of simulation steps in which the 21-label codes reached at least 50% share of the whole code population. As we can see, the time to reach domination does not depend on the probability of label reassignment to codons ($m_{c}$). A more pronounced influence is for the probability of acquiring new labels with a code ($m_{l}$) because for larger $m_{l}$ values, the domination of the codes with 21 labels happened much faster in the simulation time than for the smaller values. The impact of genetic information exchange between codes ($m_{e}$) is particularly evident because codes encoding 21 labels did not become prevalent in the population when this exchange was absent or occurred with only minimal probability, i.e., 0.01.

In contrast to that, the predominance of these codes appeared very quickly for values larger than 0.02. For 0.02, dominance was reached between 110 and 7810 (mean 796) steps, but for a probability of 0.03–0.09, the code dominated in 40–210 (mean 61) steps. The results indicate that the label addition and the exchange of genetic information (HGT) can significantly speed up the process of coding system evolution.

**Figure 7:**
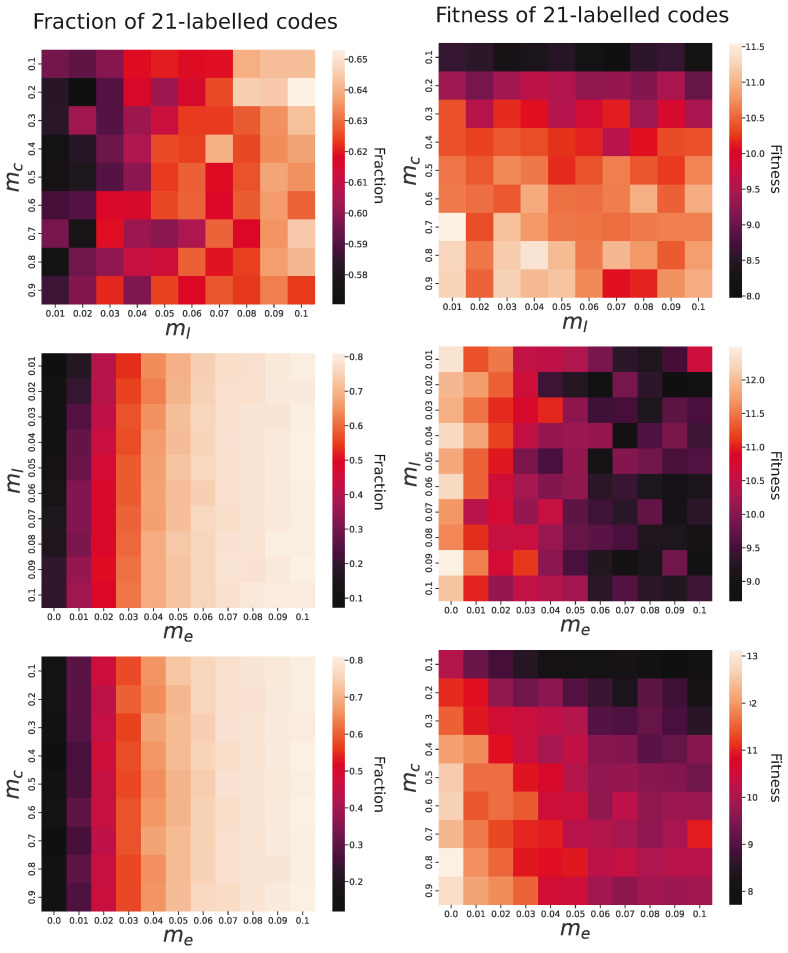
The fraction and fitness of 21-label codes in the evolving population at the end of the simulations in terms of their relationships with various combinations of three parameters: the probability of change in the assignment of labels to codons ($m_{c}$), the acquisition probability of new labels with a code ($m_{l}$), and the probability of the exchange of genetic information between codes ($m_{e}$). The bright colors indicate a higher fraction or fitness of the codes, whereas dark colors indicate their smaller fraction or fitness.

A similar influence of these parameters was found on the fraction of 21-label codes (the left panel of Figure 7). The probability of label-to-code changing ($m_{c}$) did not affect the fraction, whereas higher values of a label’s addition ($m_{l}$) caused these codes to more often dominate the population of codes. However, the most pronounced impact was exerted by the information transfer ($m_{e}$) corresponding to HGT in organisms. When the codes did not exchange information about their structure, the 21-label codes constituted only 4–25% (mean 13%) of the whole population, whereas for the highest tested probability 0.1, the codes accounted for 78–83% (mean 81%).

Interestingly, the effect of the studied parameters was different on the fitness of the 21-label codes (the right panel of Figure 7). In this case, a larger probability of label-to-codon mutation ($m_{c}$) increased their fitness at the end of simulations, whereas the probability of new label introduction ($m_{l}$) did not influence this measure. For the smallest applied value $m_{c}=0.1$, the fitness was 6.2–12.6 (mean 8.4), and for the largest value $m_{c}=0.9$, it was 8–14.5 (mean 10.8). The probability of exchanging genetic information ($m_{e}$) had a rather negative effect on the fitness because, for its largest applied probability (0.1), the fitness was on average worse (mean 9.4) than when this parameter had no effect (mean 12).

To objectively assess the influence of the studied parameters on the evolution and quality of the genetic codes, we fitted regression models describing relationships between them, as seen in Table 1. The analyses showed that the exchange of genetic information between codes ($m_{e}$) and the probability of a new label’s acquisition ($m_{l}$) were significantly different from zero and had a substantial effect on the increase in the fraction of 21-label codes recorded at the end of the simulations as well as on their first occurrence and domination over other codes, i.e., the shortening simulation time in which these codes developed and exceeded 50% contribution (Table 1). However, the impact of $m_{e}$ on the fraction was almost five times larger than $m_{l}$, but their influence on the first occurrence and domination was comparable. In addition, the coefficient at the interaction between $m_{e}$ and $m_{l}$ also turned out to be significantly different from zero. Interestingly, its sign was opposite to the coefficients at the individual independent variables. This indicates that the relationship between one of these variables and a dependent variable became weaker with the increase in another of these variables.

The exchange of information about coded labels between codes ($m_{e}$) also demonstrated significant influence on code fitness (Table 1). However, this impact was negative. On the other hand, the higher probability of change in label-to-codon assignment ($m_{c}$) significantly increased the fitness. The negative effect of $m_{e}$ was about 2.5 times stronger than the positive. Interactions between the studied variables were included in the selected model but were not statistically significant.

## 3. Discussion

The problem of the evolution of the standard genetic code has been discussed by many authors since the first decryption of codons’ meanings [8,9,10,11,12,13,43]. Among many hypotheses concerning the emergence of coding systems and decisive factors that might shape their structure, the idea of a collective evolution of many coding variants and the transfer of genetic information is very interesting [34,35]. Therefore, we simulated the evolution of many codes, assuming the genetic information exchange between them.

It seems probable that there were many trials and versions of coding genetic information before the standard genetic code evolved. Early organisms containing these codes transferred genetic information on a massive scale via horizontal gene transfer (HGT) [44,45]. This process constrained the universality of the code and could also increase the optimality of the code in terms of amino acid replacements [34,35]. The sharing of common information by organisms and possession of compatible translational machinery were profitable because these organism could obtain novel genes.

Our studies demonstrated that the HGT substantially accelerated the evolution of primordial codes to those characterized by more encoded amino acids. The increase in the probability of this process (parameter $m_{e}$) substantially shortened the emergence of 21-label codes as well as accelerated their domination and contribution among other codes. This speed-up resulted not only from multiple codes, i.e., more potential solutions, subjected to mutation and selection, but mainly from the exchange and acquisition of good code patterns that evolved independently at the same time in many early organisms. In the real past, this could occur, for example, through the transfer of genes coding for tRNAs and aminoacyl-tRNA synthetases that had already unambiguously assigned appropriate amino acids. Genes encoding components of translational machinery, including ribosomal proteins responsible for the translation and tRNA-codon recognition, were also important in the reading of genetic information. In the early stages of life evolution, HGT caused changes in codon meaning and code organization, which helped in the selection of codes encoding more amino acids. However, after the evolution of a better code, its substantial changes were deleterious due to the production of many incorrectly folded proteins and the loss of benefits from acquiring new genetic information [15]. Nevertheless, the modified codes can be advantageous for some hosts to protect them against parasite invasions due to genetic incompatibility.

The second factor that accelerated the emergence of 21-label codes and their dominance over other evolving codes was the gradual incorporation of new amino acids into the codes ($m_{l}$) but its influence was usually weaker than $m_{e}$. We started our simulation from a restricted number of coded labels, no more than seven, and observed a fast increase in their number with time. In fact, many studies have identified groups of amino acids that were added early and late to the genetic code [41,46,47]. The order of the amino acids to the code could result from their occurrence in biosynthetic pathways [48,49,50,51], the catalytic features of amino acids present in ribozymes, protein folding [52], minimizing disorders in already synthesized proteins [53], and duplications of genes coding for tRNAs and aminoacyl-tRNA synthetases associated with amino acids with similar physicochemical properties [11,15,54,55,56,57]. Undoubtedly, the inclusion of many amino acids with various properties was beneficial because it increased the diversity of synthesized proteins [10,15,53,58].

Interestingly, analyses showed that parameter $m_{e}$ negatively influenced the fitness of the codes, describing the precision of label-to-codon assignment. This adverse effect was probably caused by the exchange of coding patterns between codes that were individually and differently optimized. The interchange of coding structures likely disorganized their structures. On the biological level, we can imagine that the influx of new genetic information, e.g., genes, could disturb the already established interactions between coded products involved in reading genetic information. It required a new optimization of the whole system to incorporate the newly obtained components. Despite the temporary decrease in the fitness, in the long run, the acquisition of new information by the codes was beneficial because the coding systems could finally reach higher fitness and complexity levels. In fact, even for the largest tested value of $m_{e}=0.1$, the fitness of the 21-label codes ranged from 7.2 to 18.8 (mean 9.4) at the end of the simulations, which is much higher than the values at their first occurrence, i.e., ranging from −49.3 to 3.4 (mean −9.4).

The updated optimization could occur through gradual mutations in the components of the genetic system, which was modeled in our simulations using the third parameter $m_{c}$, i.e., the probability of modifications in the labeling of codons. This parameter corresponded to progressive changes in translational apparatus and reading genetic information. Its increase positively influenced the fitness of codes causing the coding systems to achieve more precise assignments of labels to codons.

The fitness corresponding to translational accuracy rapidly grew in the early steps of simulations, which began from the primitive codes characterized by high translational ambiguity, i.e., the assignment of several labels to the same codon with a low or moderate probability. At the molecular level, it can be associated with the inaccurate loading of amino acids by aminoacyl-tRNA synthetases onto tRNAs and incorrect interaction between codons and tRNA anticodons during translation. It was postulated that the primordial codes were characterized by the high translational noise and error, which was reduced during the code evolution [9,36], as our simulations demonstrated. The transformation from ambiguous to unambiguous codes could occur via a 2-1-3 model [25,59] or the four-column theory [53], in which the second codon position was responsible for distinguishing encoded amino acids at the early stage of code evolution, whereas the first codon position—and later the third—were incorporated into the recognition process over time. The assumption is included in our model in different types of inaccuracies in codon reading ($N_{1}$, $N_{2}$, and $N_{3}$) because they assume that a coded label had one or two fixed codon positions identical to a reference codon. The reduction in the imprecise codon meaning could also be reduced by the duplication of tRNA genes and chemical modification of tRNA molecules because the larger and more diversified population of tRNAs could enable more efficient matching of amino acids to the coding system.

However, the evolution of the SGC could be more complex. It was proposed that a primordial code called operational RNA code was based on aminoacyl-tRNA synthetases that did not recognize anticodons but RNA minihelices (proto-tRNAs) corresponding to the modern tRNA’s acceptor stems [60,61,62]. Thus, this code was initially responsible for protein synthesis, involving at least some amino acids (tyrosine, serine, and leucine), whereas the current anticodon-based coding system evolved later together with the evolution of the synthetases and tRNAs acquiring additional structural and functional domains. The emergence of this code would shorten the time necessary for the integration of amino acids into the final code (in our model described by $m_{l}$). The components of the operational code could have also been subjected to horizontal gene transfer (in our model described by $m_{e}$), which further accelerated the initial SGC evolution.

To summarize, the genetic codes evolved via simulations acquired new information and developed more accurate assignments of labels to codons. Unlike in our previous study [37], where coding systems were constrained to encode an equal number of labels, the present model allowed for variability in both the amino acid repertoire and coding system types. As a result, evolution was enriched by the coevolution of genetic codes encoding different numbers of labels. Furthermore, the population of primitive genetic codes was influenced by three key factors: (i) changes in the assignment of encoded labels (i.e., amino acids and stop signals) to codons ($m_{c}$), (ii) the gradual introduction of new labels ($m_{l}$), and (iii) the exchange of genetic information between coevolving codes ($m_{e}$). Parameters $m_{l}$ and, in particular, $m_{e}$ significantly accelerated the transition from simpler codes with fewer labels to those encoding the full set of 21 translational signals. Although genetic information transfer initially disrupted coding system fitness, this effect was counterbalanced by $m_{c}$, which enabled the emergence of codes with increasingly unambiguous codon meanings. Our simulations demonstrated that 21-label codes could arise under various parameter combinations, suggesting multiple potential evolutionary pathways from primitive to more advanced genetic codes. However, the question of which pathway ultimately led to the emergence of the SGC remains open.

## 4. Materials and Methods

The applied simulation procedure was based on evolutionary algorithms, which are biologically inspired techniques generally exploited in optimization tasks when analytical solutions do not exist or are computationally infeasible. Therefore, this approach is appropriate for studying the evolution of coding systems with the many parameters of unknown or complex relationships and interactions.

**Figure 8:**
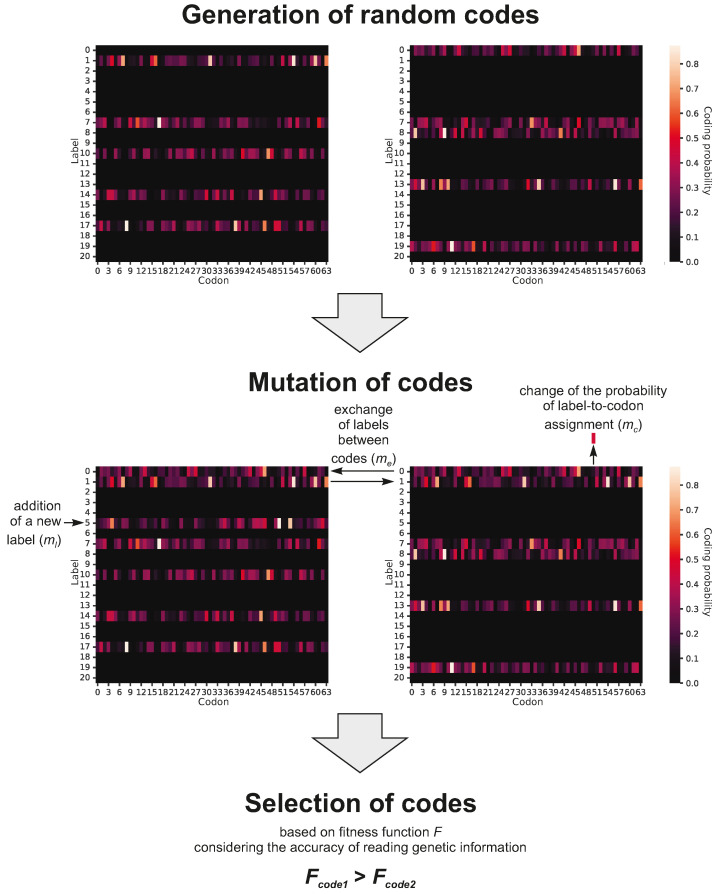
The scheme of the algorithm used in the simulation of the genetic code evolution. Two example codes, each with five labels, are shown.

The simulations began with a set of codes represented by a matrix encoding a small number of amino acid ambiguously assigned to codons (Figure 8). The number of coded labels could increase through the stepwise addition of new amino acids into the codes, whereas the assignment could change due to mutation and the exchange of information between coevolving coding systems. These codes were subjected to a selection process for the accuracy of reading genetic information and increasing coding potential, which was described by a fitness function. The mutation and selection processes were modeled by appropriate genetic operators. We predefined 10,000 steps as the duration for all simulations, as this number was sufficient to ensure the stabilization of the simulated parameters.

### 4.1. Genetic Code Representation

The simulations were conducted on a population of 1000 genetic codes (individuals). The initial coding systems were generated randomly according to the truncated Poisson distribution with λ=3, assuming that the maximum number of encoded labels, i.e., amino acids and stop coding signal, did not exceed seven. The initial primitive codes were characterized by a high ambiguity in the assignment of labels to codons. Mathematically speaking, each genetic code is represented as a matrix $P=p_{cl}$, where *c* denotes codons for 1≤c≤64, and *l* denotes the encoded labels for 1≤l≤21. Thereby, the matrix consists of 64 columns, i.e., codons, and 21 rows representing labels. Each column in the matrix P contains a probability distribution function, which assigns 21 possible labels to 64 codons. Therefore, values in the matrix column sum up to one. As a consequence, $p_{cl}$ is the probability that codon *c* encodes label *l*. In the case of the initial random codes, a single label was assigned to multiple codons with low to moderate probability. However, in the optimized codes, the label was coded with a much higher probability by a smaller number of codons. The extreme case in this representation is the standard genetic code, in which a given codon encodes only one label with probability one.

Figure 2 presents a graphical representation of a primitive genetic code encoding only five labels. Each cell of the matrix has ascribed a value of coding probability P represented by respective color intensity. The presented code is at the early stage of evolution because it includes only five labels encoded with non-zero probability. The rest of the 16 rows corresponding to other labels are black because they are coded by no codon and represent the space for future code extensions.

**Figure 9:**
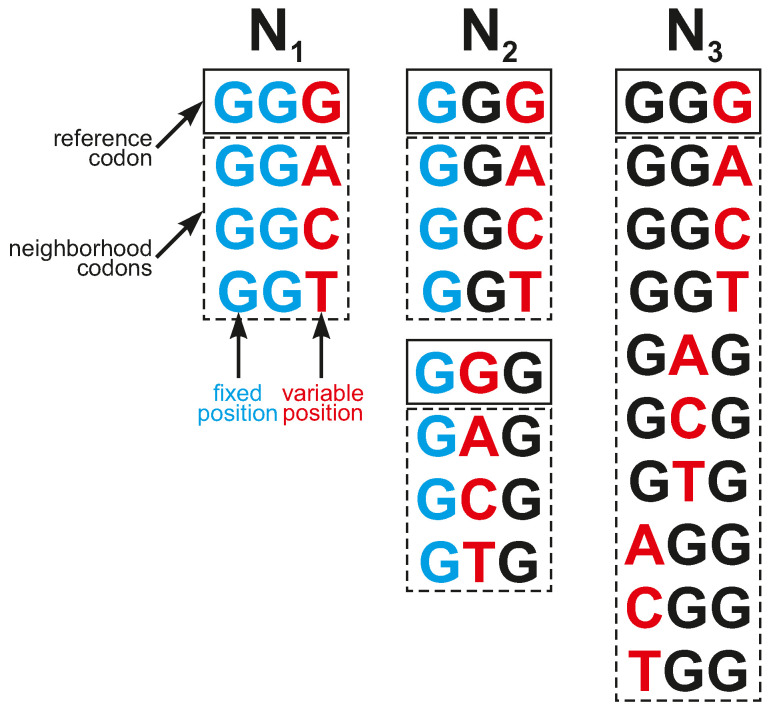
The three types of codon neighborhoods, $\left(N_{1}\right)$, $\left(N_{2}\right)$, and $\left(N_{3}\right)$, describing inaccuracies in codon reading and applied in the simulation.

The genetic codes were characterized by various types of reading systems, which represented differences in codon recognition. These differences and their impact on the structure of genetic code were discussed previously [37,63]. Simply speaking, all possible inaccuracies in codon assignment were defined by specific codon neighborhoods, i.e., the group of codons, which together with the reference codons *c* encoded selected labels. They were used to model different types of inaccuracies in codon reading. We considered three types of codon neighborhoods (Figure 9):

*N*_1_ —All codons belonging to a given group and encoding a fixed label had two fixed codon positions identical to codon *c* and differed in exactly one nucleotide at another codon position;
*N*_2_ —All codons belonging to a given group and encoding a fixed label had one fixed codon position identical to codon *c* and differed in exactly one nucleotide in one of other two codon positions;
*N*_3_ —All codons belonging to a given group and encoding a fixed label differed in exactly one nucleotide to codon *c* in any codon position.

Thus, all codons from a given group differed in at most one nucleotide position from the reference codon, and the size of the codon neighborhoods including the reference codon was four for $\left(N_{1}\right)$, seven for $\left(N_{2}\right)$, and ten for $\left(N_{3}\right)$. Codon group $N_{1}$ corresponds to the organization of the SGC, whereas $N_{2}$ and $N_{3}$ are its generalizations.

### 4.2. Genetic Operators

The mutation operator was necessary for the evolution of simulated genetic codes because it introduced random modifications into the coding systems. The mutation part is composed of three independent processes. They were responsible for changing the probability of label-to-codon assignment ($m_{c}$) and expanding genetic information, i.e., including new amino acids into the coding repertoire ($m_{l}$) as well as information exchange between codes ($m_{e}$). We applied the following values for these parameters: the probability of label-to-codon mutation $m_{c}=0.1,0.2,\ldots ,0.9$; the probability of new label introduction into a code $m_{l}=0.01,0.02,\ldots ,0.1$; and the probability of information exchange between codes $m_{e}=0,0.01,0.02,\ldots ,0.1$. The information transfer was modeled by exchanging the whole labels with their coding probabilities, i.e., rows in the matrix, between the codes. It should be noted that after all code modifications and changing the probabilities of coding labels by the codons, each column in every code matrix was normalized to sum to one. We ran the simulations under all possible combinations of these parameters. In the case of $m_{l}$ and $m_{e}$, we assumed values up to 0.1 because the initial simulations showed that the 21-label codes were very quickly generated above this value, and increasing their values did not have an influence on the results. Instead of applying the large values, we decreased the increment in the values to test more values. We ran 990 different simulations in total.

### 4.3. Fitness Function

The selection operator was responsible for assessing the quality of genetic codes using a fitness function *F*, which was based on the formula introduced in [37,63]. Equation (1) presents this function, computed for a genetic code that encodes *L* labels.

(1) $$F=log(\sum_{c_{1}^{\prime }\in N_{j_{1}}\left(c_{1}\right),\ldots ,c_{l}^{\prime }\in N_{j_{L}}\left(c_{L}\right)}P\left(l=1|c_{1}^{\prime }\right)P\left(j_{1}\right)P\left(l=2|c_{2}^{\prime }\right)P\left(j_{2}\right)\cdot \ldots \cdot P\left(l=L|c_{L}^{\prime }\right)P\left(j_{L}\right))$$

Each label *l* is coded by three types of neighborhoods $j_{1},j_{2},\ldots ,j_{L}$, where $j_{i}=1,2,3$ around reference codons $c_{1},c_{2},\ldots ,c_{L}$. These neighborhoods are defined according to the $N_{1},N_{2}$, and $N_{3}$ reading models. In this context, $P(j_{1})$ is the probability of choosing the $N_{1}$ reading model for codon $c_{1}$, whereas $P(l=1|c_{1}^{\prime })$ is the probability that a codon $c_{1}^{\prime }$ encodes label $l_{1}$. Therefore, *F* is in fact a logarithm over the sum of all possible products of probabilities that a given codon $c_{i}^{\prime }\in N_{j_{i}}$ encodes given labels l=1,2,…,L. The value of *F* calculated for a given genetic code includes information about the total number of labels and the type of reading systems. Generally, codes with higher values of *F* are characterized by higher coding probability for a selected number of labels and are more likely to be selected for the next generations.

The *F* function is proportional to the total probability that a coding system encodes all studied labels. Moreover, the value of the *F* function increases as all codons tend to unambiguously encode respective labels. Therefore, the coding system with a higher *F* value is characterized by lower noise, i.e., less ambiguity in label assignment. If we assume that the standard genetic code represents a deterministic label-to-codon mapping, we would expect it to yield the highest *F* values. However, it should be noted that the structure of the SGC is only one of many possible configurations that can minimize coding assignment error. Consequently, there exist multiple alternative solutions that locally maximize this function.

## Figures and Tables

**Table 1:** Adjusted $R^{2}$, regression coefficients, and their significance levels (*p*-values) of models, describing the influence of three parameters, the probability of change in label-to-codon assignment ($m_{c}$), the probability of new label acquisition ($m_{l}$), and the probability of information exchange between codes ($m_{e}$), on the first occurrence and domination of 21-label codes in the whole population as well as the fraction and fitness of these codes at the end of simulation. Statistically significant parameters are in bold. The models were obtained based on the Akaike information criterion (AIC) and using function *lm* in R software (Version 4.5.1) [42].

Independent Variables and Parameters	Dependent Variables							
	First Occurrence		Domination		Fraction		Fitness	
	Coefficient	*p*-Value	Coefficient	*p*-Value	Coefficient	*p*-Value	Coefficient	*p*-Value
Intercept	**78.39**	**<2 $\times \;10^{-16}$**	**1297.8**	**<2 $\times \;10^{-16}$**	**0.22**	**<2 $\times \;10^{-16}$**	**10.19**	**<2 $\times \;10^{-16}$**
$m_{c}$	2.45	0.607	-	-	-	-	**2.94**	**1.3 $\times \;10^{-8}$**
$m_{l}$	**−560.3**	**<2 $\times \;10^{-16}$**	**−14,943.8**	**<2 $\times \;10^{-16}$**	**1.54**	**3.5 $\times \;10^{-14}$**	−4.42	0.344
$m_{e}$	**−670.0**	**<2 $\times \;10^{-16}$**	**−16,087.5**	**<2 $\times \;10^{-16}$**	**7.28**	**<2 $\times \;10^{-16}$**	**−26.82**	**5.3 $\times \;10^{-8}$**
$m_{c}:m_{l}$	−38.06	0.620	-	-	-	-	1.86	0.822
$m_{c}:m_{e}$	107.6	0.181	-	-	-	-	0.26	0.976
$m_{l}:m_{e}$	**6022.8**	**4.8 $\times \;10^{-16}$**	**191,644.9**	**<2 $\times \;10^{-16}$**	**−18.37**	**7.0 $\times \;10^{-8}$**	57.23	0.468
$m_{c}:m_{l}:m_{e}$	−994.5	0.443	-	-	-	-	−98.22	0.483
Adjusted $R^{2}$	**0.69**	**<2.2 $\times \;10^{-16}$**	**0.20**	**<2.2 $\times \;10^{-16}$**	**0.81**	**<2.2 $\times \;10^{-16}$**	**0.53**	**<2.2 $\times \;10^{-16}$**

## Data Availability

The datasets used and analyzed during the current study are available from the corresponding author on reasonable request.
